# Molecular features of aggressive thyroid cancer

**DOI:** 10.3389/fonc.2022.1099280

**Published:** 2022-12-20

**Authors:** Giusy Elia, Armando Patrizio, Francesca Ragusa, Sabrina Rosaria Paparo, Valeria Mazzi, Eugenia Balestri, Chiara Botrini, Licia Rugani, Salvatore Benvenga, Gabriele Materazzi, Claudio Spinelli, Alessandro Antonelli, Poupak Fallahi, Silvia Martina Ferrari

**Affiliations:** ^1^ Department of Surgical, Medical and Molecular Pathology and Critical Area, University of Pisa, Pisa, Italy; ^2^ Department of Emergency Medicine, Azienda Ospedaliero-Universitaria Pisana, Pisa, Italy; ^3^ Department of Translational Research and New Technologies in Medicine and Surgery, University of Pisa, Pisa, Italy; ^4^ Department of Clinical and Experimental Medicine, University of Messina, Messina, Italy; ^5^ Master Program on Childhood, Adolescent and Women’s Endocrine Health, University of Messina, Messina, Italy; ^6^ Interdepartmental Program of Molecular and Clinical Endocrinology and Women’s Endocrine Health, Azienda Ospedaliera Universitaria Policlinico ‘G. Martino’, Messina, Italy; ^7^ Department of Clinical and Experimental Medicine, University of Pisa, Pisa, Italy

**Keywords:** aggressive thyroid cancer, genetic mutations, molecular features, RAI refractioness, targeted therapy

## Abstract

Poorly differentiated thyroid cancer (PDTC) and anaplastic thyroid cancer (ATC) have a worse prognosis with respect to well differentiated TC, and the loss of the capability of up-taking ^131^I is one of the main features characterizing aggressive TC. The knowledge of the genomic landscape of TC can help clinicians to discover the responsible alterations underlying more advance diseases and to address more tailored therapy. In fact, to date, the antiangiogenic multi-targeted kinase inhibitor (aaMKIs) sorafenib, lenvatinib, and cabozantinib, have been approved for the therapy of aggressive radioiodine (RAI)-resistant papillary TC (PTC) or follicular TC (FTC). Several other compounds, including immunotherapies, have been introduced and, in part, approved for the treatment of TC harboring specific mutations. For example, selpercatinib and pralsetinib inhibit mutant RET in medullary thyroid cancer but they can also block the RET fusion proteins-mediated signaling found in PTC. Entrectinib and larotrectinib, can be used in patients with progressive RAI-resistant TC harboring TRK fusion proteins. In addition FDA authorized the association of dabrafenib (BRAF^V600E^ inhibitor) and trametinib (MEK inhibitor) for the treatment of BRAF^V600E^-mutated ATC. These drugs not only can limit the cancer spread, but in some circumstance they are able to induce the re-differentiation of aggressive tumors, which can be again submitted to new attempts of RAI therapy. In this review we explore the current knowledge on the genetic landscape of TC and its implication on the development of new precise therapeutic strategies.

## Introduction

Thyroid cancer (TC) is a highly diffuse endocrine tumor affecting especially the female gender with a low death rate but increasing worldwide (1–4). TCs classification is based on the cells of origin with an incidence that changes according to the different histotypes. The differentiated TC (DTC) is the most common tumor, which arises from thyroid follicular cells, and represents with papillary TC (PTC), and follicular TC (FTC) about 85–95% of all TCs. Hürthle cells TC and poorly differentiated TC (PDTC) account for 2–5% of all TCs, and the anaplastic TC (ATC) comprises about 1.7% of all cases of TC. Medullary TC (MTC) arises from para-follicular C cells of neuroendocrine origin, accounting for 3–5% of all TCs (5).

Patients with PDTC and ATC have a worse prognosis with respect to well differentiated TC (WDTC), and a lower overall survival (OS) rate with a mean survival of about 3.2 years and 6 months, respectively (6). High rate of disease relapse is registered in PDTC patients, who report frequent local invasion of the disease at the level of trachea and/or esophagus, and also distant progression to the liver, lungs, bone and brain (7–9).

Some PDTC tumors are characterized by refractoriness to T4-mediated TSH suppression or to the therapy with radioiodine (RAI) (7).

ATC is a very aggressive cancer usually originating from DTCs or PDTCs, and is characterized by a quickly growth that can vary from days to several weeks; it is often associated to dysphagia, acute hoarseness, dyspnea, and/or neck pain (7, 10).

Thyroid ultrasound (US) helps in stratifying the risk of malignancy of thyroid nodules, that according to their morphological features (shape, size, echogenicity, margins, the presence of microcalcifications) can be further examined by the fine needle aspiration cytology (FNAC) (11).

The criteria defining the risk of malignancy for biopsied nodules and their subsequent clinical management follow the Bethesda classification system (12).

However, it is often challenging make the right therapeutic decision with indeterminate thyroid nodules, and molecular testing of genetic mutations related to TC can improve the risk stratification supporting the decision-making process in order to avoid unnecessary invasive procedure, such as surgery, and predicting possible adverse clinical outcomes in the post-operative phase (11, 12).

## Thyroid cancer molecular alterations

Some of the genetic TC alterations are called “driver” mutations that promote the normal cell transition into cancerous one, whereas the “passenger” mutations are the consequences of carcinogenesis and of loss of differentiation (13, 14). About 90% of alterations are mutually exclusive activating oncogene *BRAF* (~60%), *RAS* (~13%), and rearrangements [ALK, RET, and NTRK genes (~5%)]; whereas the other 10% are loss-of-function of tumor suppressor genes (including PPARγ, PTEN, and TP53) (15–17). The Cancer Genome Atlas (TCGA) documented aberrations of genes in 97% of PTCs, including driver genes *CHEK2, EIF1AX*, and *PPM1D*, members of the phosphoinositide 3-kinase (PI3K) pathway and other gene fusions (17), however 3% of PTCs (called “dark matter”) still are genetically undefined (18). The molecular mechanisms that guide the progression to a more aggressive pattern are not largely elucidated (19). The genetic alterations per tumor found in ATC are higher in comparison to PTC and FTC (16); and according to TCGA, PDTC has also a higher mutational burden compared to PTC, but lower than ATC. Genomic instability in PDTC and ATC involve both somatic driver mutations and gene fusions (17, 20). Parallel sequencing studies have been carried out on both PDTCs and ATCs, in order to study their molecular features and discovering the differences between these two types of tumors. Elevated frequencies of TERT promoter, TP53, PTEN and PIK3CA mutations have been observed in ATCs with respect to PDTCs; ATCs also have NF1, NF2, ATM, CDKN2A, CDKN2B and RB1 mutations. Instead, PDTCs showed a higher frequencies of gene fusions (RET, ALK, NTRK1, NTRK3) (21). Recently, next-generation sequencing (NGS) studies, have revealed molecular clues underlying the progression of DTC to PDTC and ATC (15, 16).

## Genetic pathways, and epigenetic mechanisms implicated in TC pathogenesis and progression

Most of the TC primary driver oncogenes activate the mitogen activated protein kinase (MAPK) and the PI3K pathways (22–24); the alterations involving these pathways are the most found in ATC and PDTC (20). BRAF^V600E^ and RAS-like mutations, including three highly homologous isoforms (NRAS, KRAS and HRAS) are the most common found in TC.

According to the TCGA, BRAF^V600E^ is the most frequent driver mutations associated with PTC (1, 17, 25); found in 25% of ATC, and associated with tumor aggressiveness, and a bad prognosis (26, 27). Moreover, it is related to an absent or reduced expression of various genes, such as those encoding thyroid-peroxidase, the sodium-iodide symporter, Tg, TSH receptor, and pendrin genes (SLC26A4) (1, 28, 29). Therefore, it is suggested as a predictive marker of PTC persistence or recurrence, decreased efficacy of RAI therapy (30), and reduction of the OS (13, 31–33).

Mutated BRAF PTC has been related to different clinical-pathological conditions with a negative prognostic impact, and a more aggressive behaviour (extra-thyroidal extension, lymph node metastases, advanced disease stage) (1, 34, 35). Other studies showed no kind of correlation between BRAF^V600E^ and any of the PTC aggressiveness features (13, 36). However, the detection of BRAF^V600E^ in FNAC improves the diagnostic accuracy of PTC reducing also false-negative results (13).

RAS genes mutations are mainly found in FTC and in follicular variant PTC (FVPTC) (30-45%), in PDTC (20-40%), in a less percentage of ATC (10-20%), and also in benign follicular thyroid adenoma (20-25%), while rarely in classical PTC (1, 37). These mutations are more commonly related to indolent behaviour, follicular growth, encapsulation, and a lower incidence of nodal metastasis (11, 38). However, RAS mutations are believed to worsen TC prognosis and life expectancy inducing the passage from a WDTC to a de-differentiated type, the development of distant metastases, and recurrence (13). Furthermore, the de-differentiation effect has been supported by the chromosome instability because of mutant RAS (1, 39, 40).

Association with clinical-pathological manifestation is controversial (41, 42). Disease-specific death risk is 2.9 times higher in subjects harbouring RAS mutation with respect to those without RAS mutation (43). The detection of RAS mutation in FNAC has an important clinical meaning for indeterminate nodules, with a predictive value for cancer ranging between 74% and 88% (13).

The gradual passage or de-differentiation of WDTC to ATC it has been hypothesized to be induced by the accumulation of genetic alterations, particularly of BRAF or RAS mutations (6, 44, 45).

Point mutations have been also identified in TERT promoter, resulting in a telomerase activation that is up-regulated in 80-90% of TC; whereas it is not present in normal thyroid cells (1, 46, 47).

Duan et al. studies found that: 1) ATC with PTC components is typically characterised by a BRAF mutation, and at least one late mutation event (TP53, TERT, or PIK3CA); 2) RET fusion is more frequently associated to PDTC with PTC components. In subjects with PDTC/ATC a worse OS is related to TERT and concurrent PIK3CA mutations (6). The prognostic effect related to TERT promoter mutations was not present when BRAF mutation occurred separately, showing that the co-existence of both mutations is determinant for tumor aggressiveness (40).

Other alterations mostly found in PDTC (10-14%) than in ATC (3-5%) are the genes fusions (48). RET represents the most frequent genetic fusion, especially RET/PTC1 and RET/PTC3; NTRK, ALK and BRAF fusions are unusual (20, 48). Post-radiation exposure TC, and children, reporting or not a previously irradiation history, display a high frequency of RET/PTC1 and RET/PTC3 rearrangements. RET/PTC3 is related with the tendency for aggressive behaviour and advanced stage, higher rates of extra-thyroidal extension and lymph node metastases (13). It seems that RET/PTC is a leading mutation in thyroid carcinogenesis (49–51), it is especially related to the classic PTC subtype (51). However, according to TCGA, RET/PTC is considered a primary genetic event in only 6.8% of the PTC cohort (1, 17). The diagnostic and predictive value of RET/PTC is controversial; in fact, in cases of indeterminate cytology it is still not routinely examined by molecular testing (13, 52, 53).

As regard the NTRK1/3 rearrangements, their encoded protein is constitutively active, causing the activation of the pro-oncogenic pathways PI3K/AKT, phospholipase C (PLC-γ), and MAPK (1, 7, 54).

Also ALK, a transmembrane tyrosine kinase, when activated can trigger downstream signalling pathways, including MAPK, JAK/STAT, and PI3K/AKT. ALK gene alterations lead to disease progression and aggressiveness, and they are more detected in PDTCs, ATCs than in PTCs (1, 55).

The mechanism of age-associated genetic alterations is not still fully understood, however chromosomal rearrangements are strongly related to the exposition to ionizing radiation, while BRAF^V600E^ point mutations may be associated to excess dietary iodine intake or exposure to chemical disruptors in volcanic regions (56, 57). DNA fragility and impaired repair mechanism are both implicated in radiation-induced genetic damaged or stocastic oncogenic fusion (58–60). Young children might develop more frequently uncoupled double-stranded breaks and translocation with partner genes because they seem to be more vulnerable to the ionizing radiation activity and to the loss of the DNA repair capacity (60).

Only 2.3–2.5% of FTCs display microsatellite instability (MSI), which derived from persistent oxidative stress and subsequent impairment of DNA mismatch repair gene(s) encoding MutL-homolog DNA mismatch repair enzymes PMS1, PMS2, and MLH1, MLH3 (61–63). Since tumors harboring MSI are susceptible to anti-programmed cell death ligand 1 (PD-L1) immunotherapy, additional efforts are needed to clarify the role of mismatch repair gene deficiency in TC (64).

Epigenetic alterations influence gene expression: hyper-methylation of gene promoter sequences lead to heritable inhibition of transcription, while unmethylation results in increased gene transcription (65). Thyroid-specific tumor suppressor genes can promote cell de-differentiation if wrongly methylated during the first steps of tumorigenesis. If cell lines, with TSHR gene silenced by hyper-methylation, are treated with a demethylating agent, they can in part restore TSHR expression and subsequent TSH-induced iodine uptake and effectiveness of RAI (66–68). Other tumor suppressor genes silenced by aberrant methylation are genes encoding cyclin-dependent kinase inhibitors p15INKa and p16INK4b (69), RASSF1A (70), ECAD, RARβ-2, NIS-I, DAPK, ATM, SLC26A, SLC5A8, and TIMP3 (71, 72). It has been suggested that the latter four are associated with aggressive features (18).

## Molecular driven therapies

Several molecular driven therapies have been evaluated in aggressive TC (**Table 1**) (73). Systemic treatments for unresponsive metastatic non-anaplastic follicular cell derived TC include the antiangiogenic multitargeted kinase inhibitor (aaMKIs) sorafenib and lenvatinib (7, 23, 74–77). The Food And Drug Administration (FDA) authorized these aaMKIs, because they can improve progression-free survival as emerged from phase III randomized double blinded crossover clinical trials. Although non tested in a “head-to-head” trial, lenvatinib showed a longer progression-free median survival (18.3 months vs 3.6 months of the placebo group, p < 0.001) compared to sorafenib and to placebo group (10.8 months vs 5.8 months respectively, p < 0.0001), becoming the first-choice agent among oral aaMKIs (78, 79). Most patients demonstrated disease stabilization or minor/partial responses, which lasted mean period of 12-24 months (78). Lately, also Cabozantinib, an aaMKI previously approved by FDA for the treatment of MTC, has been authorized in case of failure of first-line therapy with lenvatinib and sorafenib, since it improves progression free survival as a second-line agent (80). These compounds do not require mutation profiling of the tumors and they can be also administered when specific targetable mutation (eg, *NTRK*, *ALK*, *RET*, or *BRAF*) have not been identified. As they target primarily the angiogenic vascular endothelial growth factor receptor (VEGFR) signaling, the side effects may include fatigue, hypertension, diarrhea, hand-foot skin reaction and other rashes, thyroid dysfunctions, hepatotoxicity, renal toxicity and fistula formation in the gastrointestinal tract and/or in other locations.

**Table 1:** FDA-approved therapies for thyroid cancer.

Drugs (commercial name)	Targets	Type of cancers	Ongoing/completed Trials in the last 5 years
Lenvatinib (Lenvima**®**)	VEGFR-1-2-3, FGFR1-2-3-4; PDGFRα, KIT, and RET	Poorly Differentiated/ATC	NCT04731740 (study suspended) in combination with Pembrolizumab (Pembrolizumab+Lenvatinib or Pembrolizumab+Chemotherapy)
		Locally Advanced Invasive TC	NCT04321954 (recruiting)
		RAI-R TC	NCT04858867 (recruiting)
		Stage IVB Locally Advanced and Unresectable or Stage IVC Metastatic ATC	NCT04171622 (recruiting) in combination with Pembrolizumab
		Recurrent, metastatic RAI-R DTC.	NCT03573960 (Active, not recruiting)
		In bone-predominant metastatic RAI-R DTC	NCT03732495 (recruiting) in combination with Denosumab
		Radioactive Iodine-Sensitive DTC	NCT03506048 (terminated) (Study has been abandoned for lack of accrual)
Sorafenib (Nexavar**®**)	BRAF, ^V600E^BRAF, c-KIT, FLT-3, CRAF, VEGFR-2; VEGFR-3, PDGFR-β	ATC	NCT03565536 (recruitment status unknown)
		TC	NCT03630120 terminated (Lack of efficacy) in association with Lenvatinib; Cabozantinib or Vandetanib for MTC
Cabozantinib (Cabometyx**®**)	MET, VEGFR, GAS6, RET, ROS1, TYRO3, MER, KIT receptor, TRKB, FLT3, TIE-2	Advanced DTC	NCT03914300 (Active, not recruiting) in combination with Nivolumab and Ipilimumab
		RAI-R DTC	NCT03690388 (Active, not recruiting)
		Advanced and progressive tumors from endocrine system (ATC, etc)	NCT04400474 (recruiting) in association with atezolizumab
		Advanced Cancer and HIV	NCT04514484 (recruiting) in association with nivolumab
Selpercatinib (Retsevmo®)	RET, VEGFR1-3, FGFR-1-2-3	Progressive, Advanced, Kinase Inhibitor Naïve, RET-Mutant MTC	NCT04211337 (recruiting)
		Advanced Solid Tumors including RET Fusion-positive Solid Tumors, MTC and other Tumors with RET Activation	NCT04280081 (Active, not recruiting)
		RET-Altered TC	NCT04759911 (recruiting)
		Pediatric Patients With Advanced RET-Altered Solid (MTC, PTC, etc) or Primary Central Nervous System Tumors	NCT03899792 (recruiting)
Pralsetinib (Gavreto**®**)	RET	RET-Mutated MTC	NCT04760288 (Not yet recruiting)
		Unresectable or Metastatic NSCLC or MTC	NCT04204928 (Approved for marketing)
Entrectinib (Rozlytrek**®**)	TRKA, TRKB, TRKC, ROS1, and ALK	Solid Tumors Harboring NTRK 1/2/3 (Trk A/B/C), ROS1, or ALK Gene Rearrangements (Fusions) (PTC, etc)	NCT02568267 (recruiting)
Larotrectinib (Vitrakvi**®**)	TRKA, TRKB, TRKC	Solid Tumors Harboring NTRK Fusion	NCT02576431 (recruiting)
		Advanced Refractory Solid Tumors, Lymphomas, or Multiple Myeloma	NCT02465060 (recruiting) MATCH Screening Trial
Dabrafenib (Tafinlar**®**) Trametinib (Mekinist**®**)	RAF kinase MEK	Locally Advanced or Metastatic, RAI-R BRAF^V600E^ Mutation-positive DTC	NCT04940052 (recruiting)
		ATC	NCT04238624 (recruiting)
		RAI-R TC	NCT05182931 (recruiting)
		RAI-R TC	NCT04554680 (recruitment status unknown)
		Metastatic TC	NCT04619316 (recruiting)
		BRAF-positive ATC	NCT04739566 (recruiting)
		BRAF Mutated ATC	NCT03975231 (recruiting) in association with IMRT
		BRAF Mutated ATC	NCT04675710 (recruiting) in association with Pembrolizumab
		RAI-R TC	NCT04544111 (recruiting) in association with PDR001
Trametinib (Mekinist**®**)	MEK	Advanced Solid Tumor Patients with a BRAF V600 Mutation	NCT05275374 (not yet recruiting) in combination with XP-102
Ipilimumab (Yervoy**®**)	anti-CTLA-4	Relapsed or Refractory Ovarian Cancer, Triple Negative Breast Cancer (TNBC), ATC, Osteosarcoma, or Other Bone and Soft Tissue Sarcomas	NCT03449108 (recruiting) in association with Nivolumab and other drugs
Nivolumab (Opdivo**®**)	anti-PD-1	Metastatic RAI-R BRAF V600 Mutant TC	NCT04061980 (recruiting) Encorafenib and Binimetinib with or without Nivolumab
		Advanced Solid Tumors (PTC, etc)	NCT04731467 (recruiting) in combination with CM-24
Pembrolizumab (Keytruda**®**)	anti-PD-1	Metastatic or Locally Advanced Anaplastic/Undifferentiated TC	NCT05119296 (recruiting)
		Poorly Chemo-responsive Thyroid and Salivary Gland Tumors	NCT03360890 (recruiting) in combination with Docetaxel
		DTC	NCT02973997 (Active, not recruiting) in combination with Lenvatinib
		ATC	NCT05059470 (recruiting)
		Malignant Neoplasms of Thyroid and Other Endocrine Glands, and other malignant cancer	NCT03435952 (recruiting) in association with Clostridium Novyi-NT and Doxycycline
		Advanced/Metastatic Solid Tumors (TC, etc)	NCT04234113 (recruiting) in combination with SO-C101
Atezolizumab (Tecentriq**®**)	anti-PD-L1	Advanced Solid Tumors (TC, etc)	NCT05253053 (recruiting) To Evaluate Efficacy and Safety of TT-00420 as Monotherapy and Combination
Selumetinib (Koselugo**®**)	MEK 1/2	Malignant Neoplasms of Thyroid and Other Endocrine Glands, and other Malignant Neoplasms	NCT03162627 (active, not recruiting) The most recently 2017 in combination with Olaparib

°All the cited trials have been obtained from the site: https://clinicaltrials.gov.

ALK, Anaplastic lymphoma kinase; ATC, Anaplastic thyroid cancer; CTLA-4, Cytotoxic T-Lymphocyte Antigen 4; DTC, Differentiated thyroid cancer; FGFR, Fibroblast growth factor receptors; FLT3, Fms-like tyrosine kinase-3; IMRT, Intensity-Modulated Radiation Therapy; MEK, Mitogen-activated protein kinase kinase; MTC, Medullary thyroid cancer; NTRK, Neurotrophic tyrosine receptor kinase; NSCLC, Non-small-cell lung cancer; PD-1, Programmed cell death protein 1; PD-L1, Programmed Death Ligand-1; PTC, Papillary thyroid cancer; PDGFR, Platelet derived growth factor receptor; RAI-R TC, Radioiodine-refractory thyroid cancer; RTK, Receptor tyrosine kinase; TC, Thyroid cancer; TRK, Tropomyosin receptor kinase VEGFR, Vascular endothelial growth factor receptors.

On the other hand, if specific driver mutations are identified (eg, *NTRK*, *ALK*, *RET*, *BRAF*), new mutation-specific kinase inhibitor should be considered which have been FDA-approved, specifically for TCs or for any tumor type harboring the same molecular target (7, 23, 81, 82). For this reason, these compounds require the tumor mutation profiling to prove their pertinence to a specific patient. For example, selpercatinib and pralsetinib inhibit mutant RET in MTC but they can also block the RET fusion proteins-mediated signaling found in PTC and other types of tumor (such as lung cancer) as documented by enduring high partial response and several complete responses rates in Phase III trials (83, 84). These RET inhibitors appear also to be better tolerated than the aaMKIs. However, emerging over time new RET mutations can cause therapeutic resistance by blocking drugs access to the active site or through other mechanisms (85, 86). The clinical trials performed for TRK inhibitors, entrectinib and larotrectinib, have documented activity also for TC (87, 88) and they can be used in some patients with progressive RAI-resistant TC harboring TRK fusion proteins. In addition, the FDA, according to a small cohort study in which ~50% of patients had partial responses to therapy, authorized the association of dabrafenib (BRAF^V600E^ inhibitor) and trametinib (MEK inhibitor) for the treatment of BRAF^V600E^-mutated ATC (89, 90). A subgroup of patients of that cohort displayed a prolonged responses of several years (89). Based on these data, it is recommended to obtain rapid BRAF^V600E^ testing in all patients with ATC (91). Regarding *BRAF* ^V600^-mutant PTC, off-label administration of a BRAF inhibitor could be considered especially for whom aaMKI therapy is contraindicate. Furthermore, BRAF^V600E^ inhibitors have showed promising results for advanced DTC in phase II studies (92, 93). Ultimately, the activity of FDA-approved immune checkpoint inhibitors (such as anti-PD1 and anti-PDL1) is also routinely tested in TC samples and predictors of response are the detection of MSI and high mutational burden (24).

## RAI-R development and redifferentiation strategies

The loss of the capability of up-taking ^131^I is one of the main features characterizing aggressive TC. The cancer therapy with RAI is based in the exploiting of Na/I symporter (NIS). NIS is primarily regulated by TSH through the cAMP pathway, and it is necessary to transport the iodide against a concentration gradient in thyroid follicular cells to synthetize thyroid hormone (94). This mechanism is lost in case of NIS downregulation or loss of function.

RAI refractoriness can be defined by different scenarios, such as the absence of RAI uptake at the initial whole body scan (WBS) or in metastatic lesions, or the loss of the capacity to uptake RAI after a previous WBS showing avidly uptake RAI metastases; a progression of the disease in a subject who has previously received RAI, or a cumulative activity of 600 mCi of ^131^I; the presence of locally advanced disease that cannot be treated by surgery or evaluated by RAI uptake (95, 96). Genetic and epigenetic alterations in the RTK/BRAF/MAPK/ERK and PI3K-AKT-mTOR pathways underly the diminished NIS signalling/activity that lead to RAI refractoriness and to a more aggressive behaviour (97): their identification can be useful to investigate new compounds able to act against these aberrant molecular mechanisms overcoming the standard cancer therapy resistance.

*In vivo* studies in mice focused on the disruption of BRAF^V600E^-driven MAPK signaling and found an increase of the iodine uptake (29). According to these findings a clinical trial has been conducted on RAI-resistant metastatic TC subjects who had undergone a whole body I^124^ PET/CT, who were then treated with selumetinib (a MEK inhibitor) for 4 weeks, and subsequently underwent a second scan (98). A partial response has been obtained in approximately 62.5% of the treated subjects, whereas the others had stable disease over a year. It has been observed a difference in the response of the patients according to their mutational status, in fact those harboring RAS mutations responded more frequently with respect to those with the BRAF^V600E^ mutations.

Other studies have been carried out by using different drugs including BRAF^V600E^, TRK, and RET inhibitors in selected patients according to their genomic tests (58, 99–101).

A study enrolled non-genomically identified patients for first RAI therapy after surgery, who were randomly assigned in a “receiving selumetinib group” and in a “no selumetinib group” and benefits in response rates between the groups were not reported (102). The redifferentiation approach could be in the future a useful strategy to delay long-term treatment with kinase inhibitors using RAI therapy.

These results suggest the use of genomic tests for treatment decisions (24).

## Conclusion

De-differentiated TC and ATC have a worse prognosis with respect to WDTC and the loss of the capability of up-taking ^131^I is one of the main features characterizing de-differentiated and aggressive TC. The knowledge of the genomic landscape of TC can help clinicians to discover the responsible alterations underlying more advance diseases and to address more tailored therapy (103–109). In fact, to date, the aaMKIs sorafenib, lenvatinib, and cabozantinib, have been approved for the therapy of aggressive RAI-resistant PTC or FTC. Several other compounds, including immunotherapies, have been introduced and, in part, approved for the treatment of TC harboring specific mutations. For example, selpercatinib and pralsetinib inhibit mutant RET in MTC but they can also block the RET fusion proteins-mediated signaling found in PTC. Entrectinib and larotrectinib, can be used in some patients with progressive RAI-resistant TC harboring TRK fusion proteins. In addition FDA authorized the association of dabrafenib and trametinib for the treatment of BRAF^V600E^-mutated ATC (89, 90).

Tyrosine kinase inhibitors drugs can act against different altered pathways implicated in the pathogenetic process of aggressive TC. However, patients can’t have a good therapeutic response to the therapies with activation of other pathways able to evade the drugs antitumoral effect. Moreover, patients can experience important side effects that can lead to the interruption of the therapy.

New therapies strategies are under investigations, with drugs against immune checkpoint inhibitors.

A good therapy strategy is knowing the molecular pattern of each patient that could aid in the choice of right therapies avoiding the administration of ineffective drugs. A personalized therapy is the challenge of the precision medicine. This challenge can be largely support by *in vitro* drug tests performed on primary tumor cells obtained from patients, that reflect the *in vivo* behavior with a predictive positive value of 60%, and negative predictive value of 90% (110–112). Furthermore, *in vitro* studies can be performed in cells obtained by using the non-invasive technique of FNAC, without the use of surgery (113–115).

Therefore, additional studies about molecular implications involved in the development of aggressive cancer, as well as about each individual patients response to chemotherapeutics will pave the way in the battle against thyroid aggressive cancer.

## Author contributions

GE, AP, SB, GM, CS, AA, PF, SMF conceived the paper. GE, AP, SMF specifically wrote the paper and controlled references. All authors reviewed and approved the final version of the manuscript. GE and AP equally contributed as first authors.
